# Controlled mutation rates in synthetic replicators

**DOI:** 10.1038/s42004-021-00474-6

**Published:** 2021-03-09

**Authors:** Andrew J. Bissette

**Affiliations:** Communications Chemistry, https://www.nature.com/communchem

## Abstract

Molecular replication could offer insight into the fundamentals of evolution, but achieving controlled mutation is difficult. Now, a synthetic replicator that allows for simple control over its mutation rate has been reported.

Synthetic molecules capable of replication have long been proposed as minimal models for biological evolution. A potential route to mimicking evolution on the molecular level involves replicating the sequence information of an oligomer or polymer, by analogy to DNA, while allowing mutation of the sequence. However, achieving mutation in replicators based on synthetic oligomers—those not based on biomolecules such as RNA—has proven challenging. Now, Diego Núñez-Villanueva and Chris Hunter from the University of Cambridge, UK, report a system capable of introducing mutations at a controlled rate (10.1039/D0SC06770A)^1^.

Studies of oligomeric replicators typically rely on supramolecular chemistry. Monomers assemble along the template oligomer before undergoing ligation to yield a copy of the template, or its complementary sequence, depending on the specific chemistry involved. If multiple monomer species are present then, in principle, mutations in the oligomer sequence can occur in this process through random error: the ‘wrong’ monomer aligns, creating a new product oligomer. But introducing open-ended mutations to replicators based on synthetic oligomers remains a challenge, and prior work has focused on replicating only homo-oligomers. Extending replication of synthetic oligomers to heteromeric systems capable of random mutation could be simplified by controlling the rate at which mutations appear in the product strands. “Supramolecular approaches that use weak dynamic interactions are error-prone, because the monomer units are never fully bound to the template”, says Hunter, “There are all kinds of competing equilibria that interfere”.

As a first step towards controlled replication and open-ended mutation of synthetic oligomers, Núñez-Villanueva and Hunter exploit a variation on a previously-reported family of replicators that use covalent assembly of the monomers^2^. In contrast to traditional supramolecular systems, here the monomers bind to the template through copper-catalysed azide–alkyne cycloaddition to form covalent analogues of base pairs, before the new strand backbone is ligated using carbodiimide coupling. The two strands—a covalent duplex—are chemically cleaved to liberate the template and product. When the product sequence is the same as the template, the overall process can be considered as replicating sequence information. Relying on functional group reactivity, rather than molecular recognition motifs, to transfer sequence information offers distinct advantages in controlling reactivity. **“**This covalent approach opens up a number of new possibilities, such as the strategy for introducing mutations described here, which would be difficult to tackle using dynamic or non-covalent base-pairs”, says Hunter.

The key innovation reported here over prior generations of the covalent replicators is the use of isosteric monomers. This ensures that monomers that encode the same residue in the product strand as is present in the template and those that introduce a mutation behave similarly. Both will fit into the product duplex interchangeably, and hopefully avoid biasing the reactivity of neighbouring residues with other monomers. As a result, the rate at which mutations in the product oligomer sequence arise can be controlled simply by varying the monomer composition: the distribution of products between direct copies, reciprocal copies, and heteromeric oligomers with one or more mutations is determined directly by the ratio of monomers. At higher mole fractions of the mutant monomer, the product distribution shifts towards reciprocal copies and heteromeric strands containing multiple mutant residues. The experimental product distributions agree well with those predicted from models assuming statistical incorporation of the monomers.

The study serves as a proof of principle for the controlled introduction of open-ended random mutations into synthetic self-replicating systems. Future development, including extension to longer, more analytically challenging oligomers, may offer a powerful complementary approach to studies of non-enzymatic nucleotide synthesis and related models for chemical evolution Fig. 1.

**Fig. 1:**
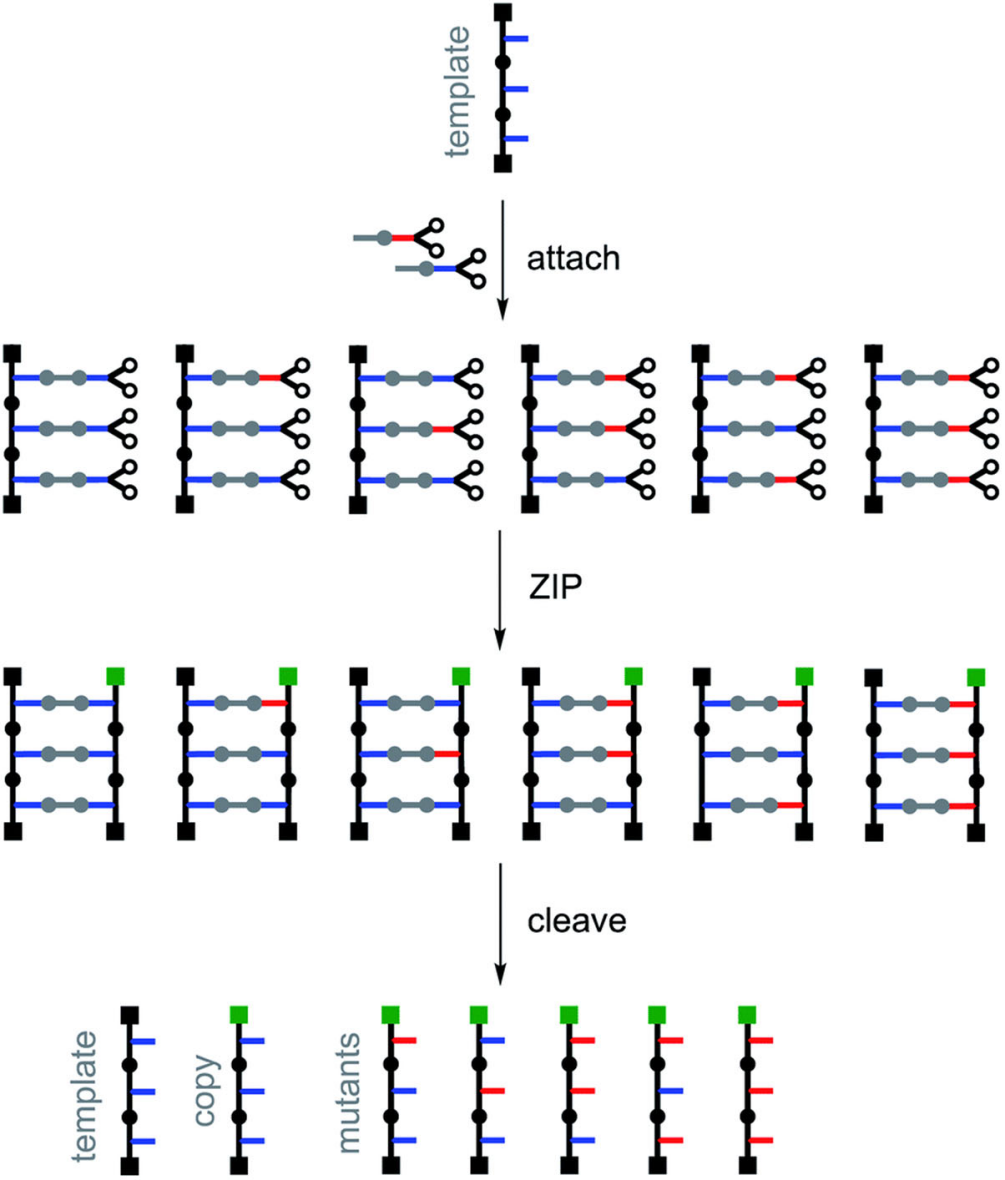
Controlled mutation in a synthetic replicator. The use of isosteric monomers in a replicator based on covalent inter-strand interactions allows for the rate of introduction of sequence mutations to be controlled solely by tuning the monomer composition. Reproduced from *Chem. Sci*. 2021, 10.1039/d0sc06770a, licensed under CC-BY 3.0.

## References

[1] Núñez-Villanueva, D. & Hunter, C. A. Controlled mutation in the replication of synthetic oligomers. *Chem. Sci*. 10.1039/d0sc06770a (2021). 10.1039/d0sc06770a PMC8179503 34163677

[2] Núñez-Villanueva D, Hunter CA (2019). Molecular replication using covalent base-pairs with traceless linkers. Org. Biomol. Chem..

